# Origin and evolution analysis and genetic characteristics of echovirus 9 in China

**DOI:** 10.1186/s12985-022-01820-3

**Published:** 2022-06-03

**Authors:** Fenfen Si, Tianjiao Ji, Dongyan Wang, Yong Zhang, Shuangli Zhu, Junhan Li, Wenbo Xu, Dongmei Yan

**Affiliations:** 1 WHO WPRO Regional Polio Reference Laboratory and NHC Key Laboratory for Biosafety, NHC Key Laboratory for Medical Virology, Chinese Center for Disease Control and Prevention, National Institute for Viral Disease Control and Prevention, Beijing, 102206 People’s Republic of China; 2 Beijing Fengtai District Center for Disease Control and Prevention, Beijing, 10071 People’s Republic of China; 3 Center for Biosafety Mega-Science, Chinese Academy of Sciences, Wuhan, 430071 People’s Republic of China

**Keywords:** Echovirus 9, Origin and evolution analysis, Temperature sensitivity, Recombination forms

## Abstract

**Background:**

Echovirus 9 (E9) is associated with a wide variety of diseases and medical conditions, and the clinical symptoms of sporadic cases caused by E9 often are severe. With a high global prevalence, E9 has caused multiple outbreaks worldwide. However, little is known about the genetic and geographic population dynamics of E9.

**Method:**

A total of 131 *VP1* gene sequences, including15 generated in this study and 116 obtained from GenBank, were used to coestimate time-resolved phylogenies to infer viral evolution and transmission in worldwide. Overlapping fragments representing whole genomes were amplified by reverse transcription polymerase chain reaction (RT-PCR) using specific primers. Then, we reported the genetic characteristics of fifteen E9 strains in the Chinese Mainland. Similarity plots and bootscanning analysis were used to determine recombination patterns of E9.

**Results:**

The estimated mean evolutionary rate of global E9 *VP1* gene was 4.278 × 10^−3^ substitutions per site per year (95% confidence interval [CI], 3.822 × 10^−3^/site/year to 4.710 × 10^−3^/site/year), and the common ancestor of E9 likely emerged around 1868 (95% CI, 1840 to 1892). The full-length genomic sequences of the fifteen E9 strains showed 76.9–79.6% nucleotide identity and 95.3–95.9% amino acid identity with E9 Barty strain. 11 of 15 E9 whole genome sequence present four recombination patterns, and E9 recombinants have extensive genetic exchanges in the 2C and P3 regions with other *Enterovirus* B (EV-B) circulated in China. Four of six E9 strains were temperature sensitive, and two were temperature resistant, and a comparative genomics analysis suggested that 411, 865 and 867 amino acid substitution in the P1 region was related to temperature sensitivity.

**Conclusion:**

This study highlights a persistent transmission network of E9 in worldwide, provides valuable information regarding the molecular epidemiology of E9.

## Background

E9, which belongs to the species EV-B of the *Picornaviridae* family, is a positive sense, single-stranded RNA virus [1]. E9 has been frequently associated with aseptic meningitis (AM) [2], hand, foot, and mouth disease (HFMD) [3], and acute flaccid paralysis (AFP) [4]. Less frequently, E9 is also associated with multiorgan infection that can rapidly lead to critical conditions, such as acute rhabdomyolysis [5], acute liver failure [6], acute renal failure [7], acute onset of type I diabetes mellitus [8]. Hence, E9 is associated with a wide variety of diseases and conditions, and the clinical symptoms of sporadic cases caused by E9 often are severe.

E9 has caused multiple outbreaks worldwide, including Asia (Japan [9] [10], China [11], et al.), Europe (Russia [12], England [13], Belgium [14], Spain [15]), Oceania (Australia [16]), South America (Brazil [17]), North America (Mexico [15], America [18]). The detection rate of E9 was found to be high in environmental monitoring [19]. Moreover, the susceptible population of E9 was preschool children [20], and nurseries are high-risk places for outbreaks [21], so the consequences are serious once an outbreak occurs. Therefore, it is necessary to strengthen the monitoring and research on E9, and guard against the re-outbreak of E9.

Despite its clinical impact and its high global prevalence, little is known about the genetic and geographic population dynamics of E9. Continuous molecular epidemiological surveillance is important to help identify newly emerging strains and to better understand trends in viral circulation [22]. In our previous study, we divided E9 into A-G genotypes and investigated the distribution of genotypes [23]. This study analyzed the population dynamics of E9 in viral evolution and transmission in global basing on the former study [24]. Recombination patterns of E9 were analysed in our study, and temperature sensitivity tests were used to determine the environmental transmission capacity.

## Materials and methods

### Ethics statement and sample collection

This study did not involve human participants or human experimentation. Only specimens (stool samples, throat swab samples) were collected from HFMD patients for public health purposes at the urging of the Ministry of Health, P. R. of China. Written informed consent for the use of their clinical samples was obtained from the parents of the children whose samples were analyzed. This study was approved by the second session of the Ethics Review Committee of the National Institute for Viral Disease Control and Prevention (NIVDC), Chinese Center for Disease Control and Prevention, all experimental protocols were approved by NIVDC, and the procedures were in compliant with the approved protocol.

Based on national HFMD surveillance program, clinical specimens (throat swabs, rectal swabs, herpes swabs, or stools) were collected from HFMD patients from thirty-one provinces or municipalities in mainland of China. All clinical specimens from HFMD patients were collected according to applicable standards and previously described protocols [25].

### Virus isolation and molecular typing

Viruses were isolated from original clinical specimens by propagation in human rhabdo-myosarcoma (RD) and human larynx carcinoma (HEp-2) cells by conventional methods. The cell lines were provided by the WHO Global Poliovirus Specialized Laboratory in the USA and were originally purchased from the American Type Culture Collection (Manassas, VA, USA). After complete EV-like CPE were observed, we harvested the infected cell cultures.

Viral RNA was extracted from the cell culture using a QIAamp Viral RNA Mini Kit (Qiagen, Hilden, Germany). RT-PCR was performed to amplify the partial VP1 coding region using the PrimeScript One Step RT-PCR Kit Ver.2 (TaKaRa, Dalian, China) with primers 490 and 492 (EV-B universal primers). The EV Genotyping Tool (a BLAST server) based on entire VP1 region was used for enterovirus serotyping. Finally, fifteen E9 strains were extracted from the specimen during the years 2013–2019 located in six provinces of China (Table 1) and include in this study.

**Table 1:** Detailed information of fifteen E9 strains in this study

Separation time	Province	Name of strain	Case type	Age	Gender	Subgenotype
2013	TianJin	TJ-2013-60	Mild	/	Male	D3
2013	HeNan	HeN-2013-5	Severe	4	Male	D3
2013	HeNan	HeN-2013-44	Mild	1	Male	D3
2013	HeNan	HeN-2013-66	Mild	2	Male	D3
2013	HeNan	HeN-2013-91	Severe	3	Male	D3
2013	ShaanXi	SaX-2013-18	Severe	1	Male	D3
2013	ShaanXi	SaX-2013-97	Mild	5	Male	D3
2013	HeBei	HeB-2013-65	Mild	1	Female	D3
2013	HeNan	HeN-2013-321	Severe	4	Male	D3
2014	HuBei	HuN-2014-103	Mild	1	Male	D3
2014	ShaanXi	SaX-2014-49	Mild	6	Male	D3
2014	ShaanXi	SaX-2014-85	Mild	2	Female	D3
2016	HuNan	HuN-2016-46	Mild	1	Male	D3
2019	JiangXi	JX19-806	Mild	2	Male	C2
2019	YunNan	X57	Severe	/	Male	D3

### Full-length genome sequencing

The full-length viral genome sequences were amplified by “primer-walking” strategy to close the gaps as necessary. Briefly, overlapping fragments representing whole genomes were amplified by RT-PCR using specific primers. The 5′end of the genome was amplified based on the manufacturer’s instruction with 5′-Full RACE Kit (Takara, Shiga, Japan). The 3′end of the genome was amplified using an oligo-dT primer (7500A) described in a previous study [26]. The primer pairs used for each step are listed in Table 2.

**Table 2:** PCR and sequencing primers

Primer	Primer sequence (5’-3’)	Reference
1S48	GGGGACAAGTTTGTACAAAAAAG	[26]
1R467	TCTGCTCCGCAGTTAGGATTA	This study
2R66	GGTACCTTTGTGCGCCTGTTT	This study
2R1182	TGCATCAGGAAATTTCCACCA	This study
3F894	AAATTCACCGAACCAGTCAAG	This study
3R2241	GTAGTGCGTTTGGCTAATCCA	This study
4F2049	TATTATGCACACTGGTCAGGT	This study
4R3366	CCCTACATACACAGCCCCAGA	This study
5F3018	GCCTACAGCAGCTTTTATGAT	This study
5R4431	ACGGCATTTGGACTTGAACTG	This study
6F4104	TGGCTCAAGAAATTCACAGAG	This study
6R5441	CTGGCGTTTCTTTTCATCATC	This study
7F4950	TGTGGAAAAGCTATCCAATTCA	This study
7R6420	CTTCACATAGGTCACCATTGG	This study
Ech97F	AGGTTAATGAGGCTGTCCTGGC	[27]
Ech97R	CCTGGGTTCAGATGGAATGT	[27]
Ech98F	GCTTGAATGATTCTGTTGCAAT	[27]
Ech98F	CGCACCGAATGCGGAGAATTTACC	[27]
9F7185	CCAAAGAACACCCAAGATCAT	This study
7500A	GGGGACCACTTTGTACAAGAAAGCTGGG(T)	[26]

### Phylodynamic and recombination analysis

131 entire VP1 sequences of E9 (including 116 E9 entire VP1 sequences downloaded from GenBank and 15 sequences obtained from this study) were used to construct Bayesian inference of phylogeny using Markov Chain Monte Carlo (MCMC). GTR (General Time Reversible) + G4 (Gamma distributed rate with four rate catagories) was selected as the best-fitting nucleotide substitution model calculated by JModeltest v2.0.1 software. Phylodynamic analyses were performed in the GTR + G model of nucleotide substitutions under the strict clock: Uncorrelated Log-normal setting for 80 million MCMC by the BEAST v1.8.4 software. After the maximum clade credibility tree had been constructed, convergence was assessed with effective sample size (ESS) values higher than 200 using TRACER v1.6 software. The analysis was sampled at every 10,000 states. Posterior probabilities were calculated with a burn-in of 10 million states. The analysis of collected data was conducted by Tracer v1.6, and Tree Annotator program was employed to output the results of the maximum clade credibility (MCC) tree model. FigTree program was then used to plot the MCC molecular evolutionary tree.

The P2 and P3 coding region sequences of the fifteen E9 strains were analyzed using the BLAST server to compare their identity with sequences from GenBank. Enterovirus genome sequences with a similarity higher than 85% were selected as potential recombination parents. Similarity plots and bootscanning analysis were performed by the SimPlot v3.5.1 with a 200-nucleotide window moving in 20-nucleotide steps. According to the distribution of informative sites, we determined recombination breakpoints.

### Assay of temperature sensitivity

According to the spatiotemporal separation distribution and genotypes, we selected six representative E9 stains (SaX-2013-18, HeB-2013-65, HeN-2013-321, HuN-2014-103, JX-2019-806, X57) to test the temperature sensitivity of the E9 strains. The temperature sensitivity of six E9 strains and two selected control strains (HTYT-ARL-AFP02F/XJ/CHN/2011, showing no temperature sensitivity and KS-MGTH90F/XJ/CHN/2011, showing temperature sensitivity) were assayed on monolayer RD cells in 24-well plates. The 24-well plates were inoculated with 50μL of undiluted virus stock solution. The plates were incubated at different temperature conditions, i.e. the optimum temperature of 36 °C and the supra-optimal temperature of 39.5 °C for virus propagation. After adsorption at 36 °C or 39.5 °C for 1 h, the unabsorbed virus inoculum was removed and 100 μL of maintenance medium was added to each well. The plates were continuously incubated at 36 or 39.5 °C and harvested respectively at five time points after infection (4, 8, 16, 24, and 48 h). The CCID50 was calculated by end-point dilution method on monolayer RD cells in 96-well plates at 36 °C. Virus isolates showing more than 2-logarithm reduction in titer at different temperatures were considered temperature-sensitive.

## Results

### O*rigin and evolution analysis and population dynamics of E9*

**Fig. 1:**
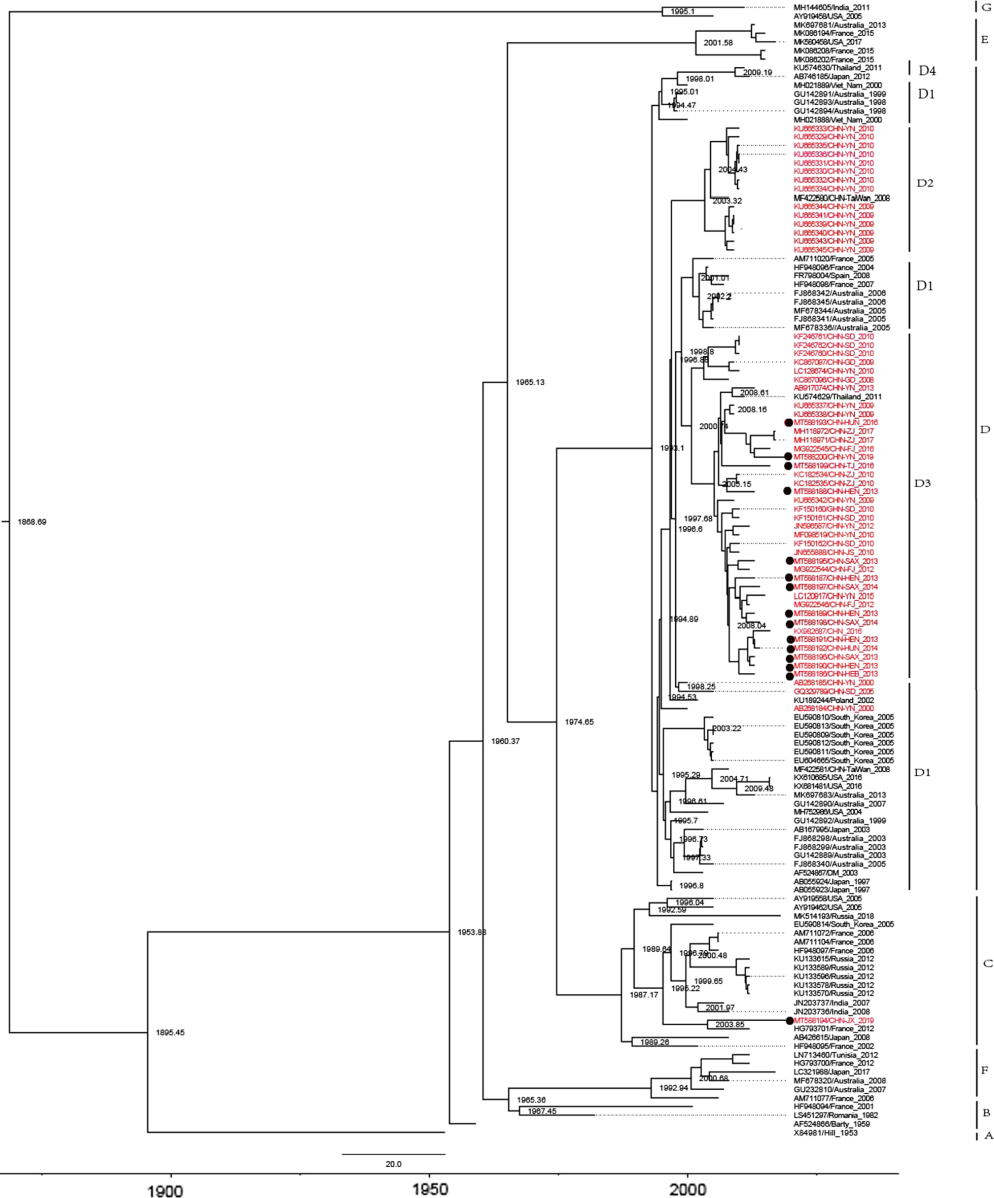
The MCC phylogenetic tree generated using the MCMC method based on the complete VP1 sequences of 121 E9 variants and the Chinese isolates was marked in red. The scale bar represents time in years. The tree was node-labelled with inferred dates of lineage splits. Each genotype is noted on the right

Bayesian phylogenetic analysis is to reconstruct time‐scaled phylogenies which can better reflect the actual virus origin and evolution. Bayesian phylogenetic analysis was performed based on E9 VP1 sequence alignment (N = 131). The results showed that the estimated mean evolutionary rate was 4.278 × 10^−3^ substitutions per site per year (95% CI, 3.822 × 10^−3^/site/year to 4.710 × 10^−3^/site/year). The common ancestor of E9 likely emerged around 1868 (95% CI, 1840 to 1892) (Fig. 1). Genotype A only includes the prototype Hill strain, whose origin can be traced back to 1895. After half a century of transmission, it was first isolated from the feces of healthy children in 1953. The origin of genotypes B, C, D, E and F can be traced back to 1953, they have the same ancestor with genotype A. It is speculated that genotypes B–F were evolved from genotype A. The origin of genotype C could date back to 1987. Significantly, all E9 strains isolated from Russia were clustered into genotype C, and the origin of these E9 strains could date back to 1989, which suggested E9 had been circulating in Russia for about 20 years before the outbreak of aseptic meningitis caused by E9 [12]. Genotype D was the most common genotype in worldwide, the origin of genotype D could date back to 1993. In 2003 and 2000, D2 and D3 sub-genotypes were formed respectively. D2 and D3 sub-genotypes are the dominant genotypes of E9 in Chinese mainland. In D2 sub-genotype, the Taiwan strain of E9 was firstly isolated in 2008, the rest were all isolated from Yunnan, China. It is suggested that D2 sub-genotype of E9 may be originated from Taiwan, China, and transmitted in Yunnan, China. Only one strain of E9 was isolated from Thailand, others were Chinese mainland strains in D3 sub-genotype. The time of origin of the D3 sub-genotype in mainland China was earlier than that in the Thailand strain, we speculated that the Thailand strain of E9 was imported from China and was not transmitted in Thailand. The origin of genotype E and F can be traced back to 2001 and 1992. Genotype G was formed by the evolution of ancient ancestors in 1995.

**Fig. 2:**
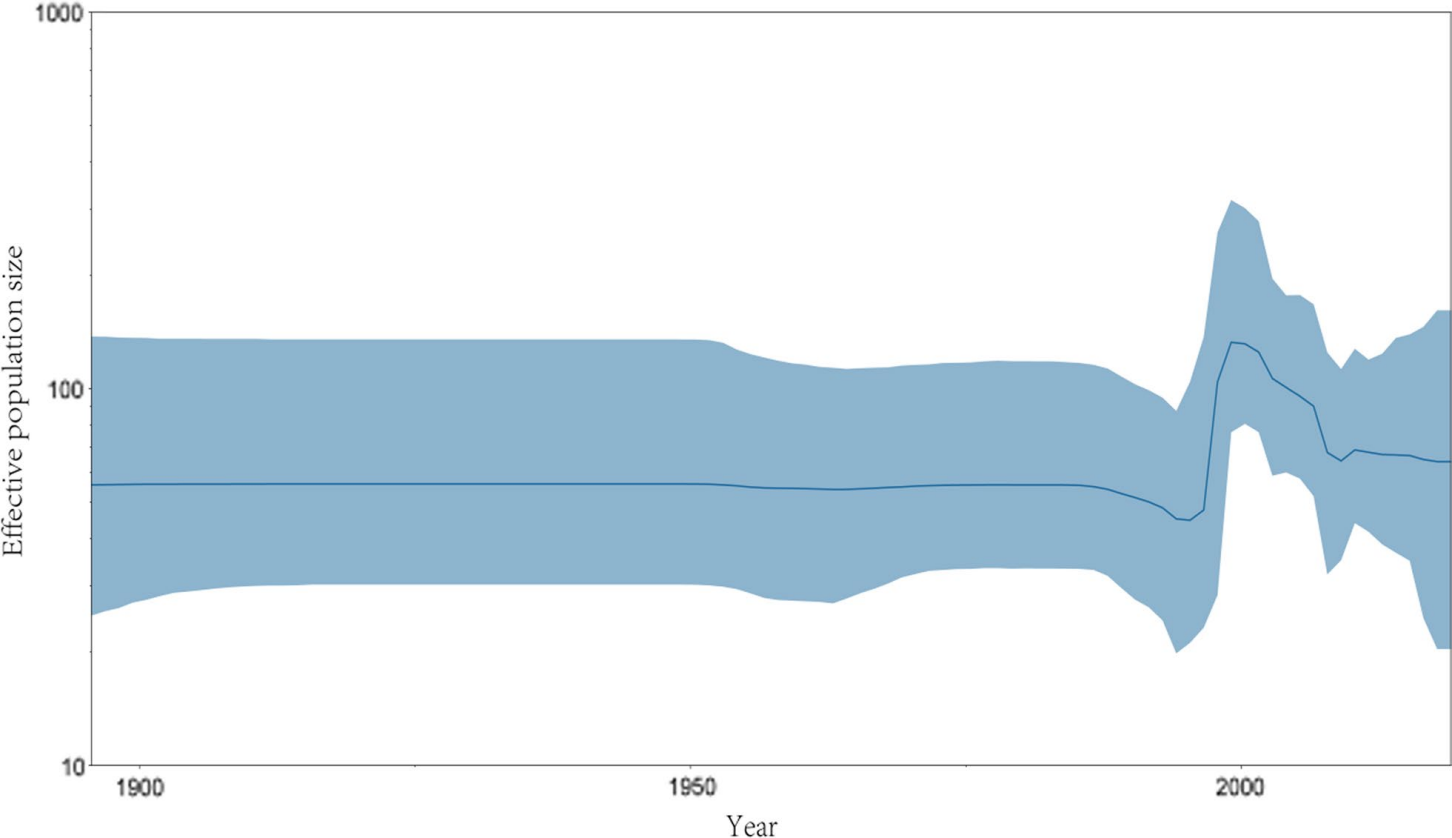
Bayesian Skyline Plots of E9

Bayesian skyline plot analyses were performed to reconstruct the past population history of E9 by measuring the dynamics of *VP1* effective population size over time. There was no obvious amplification in the population of E9 before 1990s, which maintained at a stable level. A burst of the skyline plot was observed in 1990s-2010s, probably because of faster and increasing branch of evolution of E9 at that time which were accordance with repeated E9 outbreaks reports in that time [9, 12, 18]. And after 2010s, the effective population size is to a certain level and remained stable up to now (Fig. 2).

### Full-length genome analysis of the fifteen Chinese E9 strains

The whole genome sequence of the fifteen Chinese E9 strains was determined to be 7454–7456nt long. The ORF of the fifteen Chinese E9 strains was 6609nt in length, encoding a polypeptide of 2203 amino acids, with a 741–743nt 5’-UTR and a 103-105nt 3’-UTR. The overall nucleotide composition of the fifteen strains was as follows: 27.8–28.5%A, 23.6–24.2%T, 23.0–23.6%C, and 24.2–25.0%G.

The prototype strain Hill of E9 (E9/Hill) was isolated from the rectal swab of a healthy child in 1953 and shown to be nonpathogenic in newborn mice. In contrast, strain Barty (E9/Barty) isolated from a child suffering from aseptic meningitis, are highly virulent in newborn mice [28]. Unlike Hill strain, Barty strain contains a C-terminal extension to the capsid protein VP1 with a argnine-glycine-aspartic acid (RGD) motif [29]. In previous studies, researcher considered that RGD motif was a significant factor affecting pathogenicity of E9 strains [28]. Alignment of the fifteen E9 strains in this study with strain Barty and the prototype Hill strain found that all isolates exhibited the VP1 extension with a RGD motif. We concluded that the pathogenicity of the fifteen E9 strains in this study was similar to that of strain Barty.

Then we aligned the fifteen E9 strains in this study with E9 Barty strains (Barty/X92886). The full-length genomic sequences of the fifteen E9 strains showed 76.9–79.6% nucleotide identity and 95.3–95.9% amino acid identity with E9 Barty strains. The nucleotide sequence and amino acid sequence similarities of the P1 region with Barty strains was 79.2–80.6% and 95.1–95.7%, respectively. The nucleotide sequence and amino acid sequence similarities of the fifteen E9 strains with Barty strains were 74.0–77.2% and 75.8–78.5%, 72.4–78.3% and 74.2–79.8% in the P2 and P3 region, respectively. However, fifteen E9 strains have higher homology with other EV-B prototype strains than E9, suggesting that recombination might occur in these coding regions.

### Phylogenetic analysis of the fifteen E9 strains compared with other EV-B genomes

**Fig. 3:**
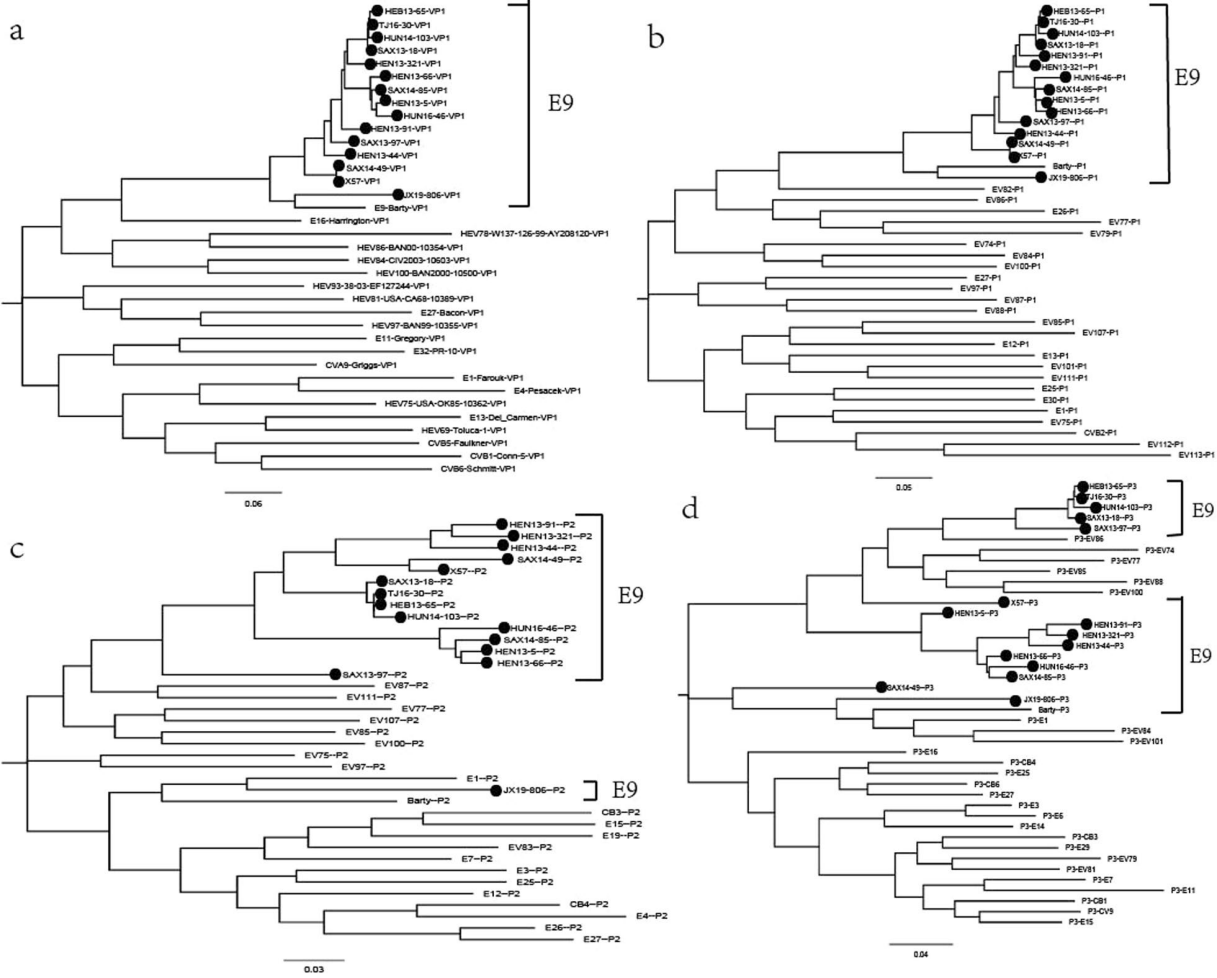
Maximum likelihood phylogenetic trees were illustrated in (**a**–**d**) based on the VP1, P1, P2, and P3 coding regions of the prototype sequence of all EV-B in the GenBank database and the fifteen Chinese E9 strains in this study. Strains isolated from this study were marked with black circles. The scale bars indicate the substitution per site per year

Maximum likelihood phylogenetic trees were constructed based on the VP1, P1, P2, and P3 coding region nucleotide sequences of the prototype sequence of all EV-B in the GenBank database and the fifteen Chinese E9 strains in this study (Fig. 3). The VP1 and P1 phylogenetic trees indicated that the fifteen E9 strains with the prototype of E9 (Barty strains) were clustered in single clade as expected. Unlike the VP1 and P1 phylogenetic trees, the phylogenetic trees based on the P2 and P3 coding regions showed that the fifteen E9 strains did not cluster in single clade and shared higher similarity with the prototype sequence of other EV-B strains than Barty strains. In P2 coding region, JX19-806 shared the highest similarity with the prototype sequence of E1. In P3 coding region, HeB13-65, TJ16-30, HuN14-103, SaX13-18, X57 and SaX13-97 were clustered with EV86, and JX19-806 was clustered with E1, EV-B84, EV101. These results suggest that the fifteen Chinese E9 strains may experience different recombination patterns in the evolutionary process.

### Four recombination patterns of the fifteen E9 strains

Potential evidence of recombination in the genome of the fifteen E9 strains was investigated via similarity plot and boot scanning analysis. The fifteen E9 strains had no significant recombination with prototype strains of other EV-B. Then, the P2 and P3 regions of the fifteen E9 strains were used to screen closely sequences available which have a strong possibility to occur recombination event in GenBank using BLAST from NCBI. All closely sequences which showing over 85% similarity with the query sequence were downloaded from GenBank.

Similarity plot and boot scanning analysis showed that the other 11 E9 strains had recombined except for HeN-2013-44, HeN-2013-91, HeN-2013-321 and JX-2019-806. We found four recombination patterns of the fifteen E9 strains named respectively RF1-RF4 via. To definitively illustrate the recombination, four groups were defined as A-D groups respectively based on the recombination forms RF1-RF4 used as the query sequence.

**Fig. 4:**
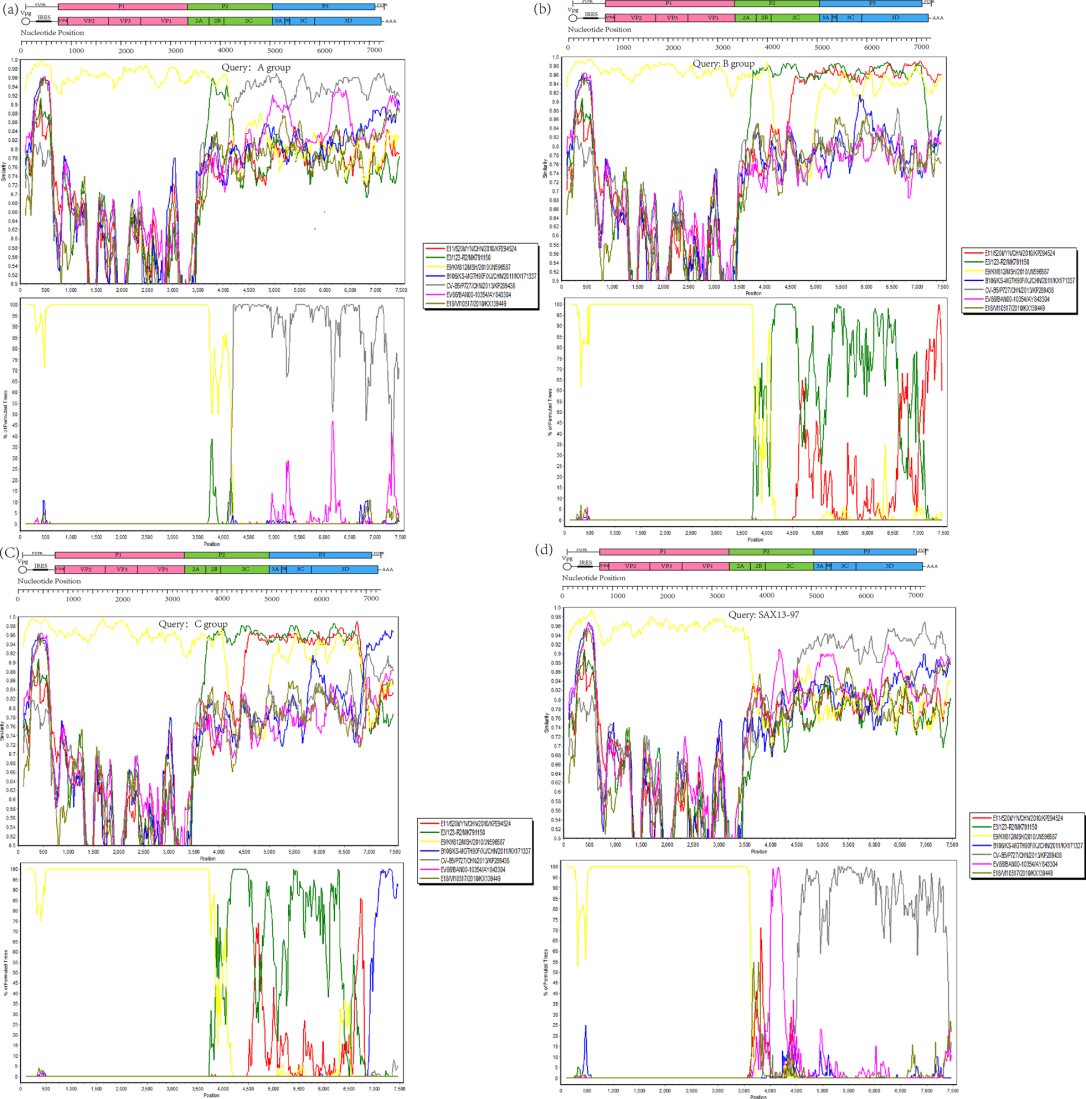
Similarity plot and bootscanning analysis of 4 types of recombination forms of the fifteen E9 strains

In the 5’-UTR and P1 region, all the fifteen E9 strains shared the highest similarity with the E9 strain isolated from Yunnan of China in 2010(KM812-JN596587). However, in the 2C-3D coding region, the query strains shared the highest identity with other serotype strains. In the 2C coding region, RF1 possibly recombined with the CVB5 strain (P727/CHN/2013/KP289438), RF2 and RF3 recombined with the E3 strain (123R2/CHN/2018/MK791150) with a high supporting value, RF4 possibly recombined with the EV-B86 strain (BAN00-10,354/USA/AY843304). In the P3 regions, RF1 and RF4 recombined with the CVB5 strain (P727/CHN/2013/KP289438) with a higher probability. RF2 and RF3 recombined with the E3 (123R2/CHN/2018/MK791150) strain in the 3A-3C regions and recombined with E11(520 k/CHN/YN/2010/KP294524) and EV-B106(KS-MGTH90F/CHN/XJ/2011/KX171337) respectively in the 3D region (Fig. 4).

### Environmental tolerance of the six China E9 strains

**Fig. 5:**
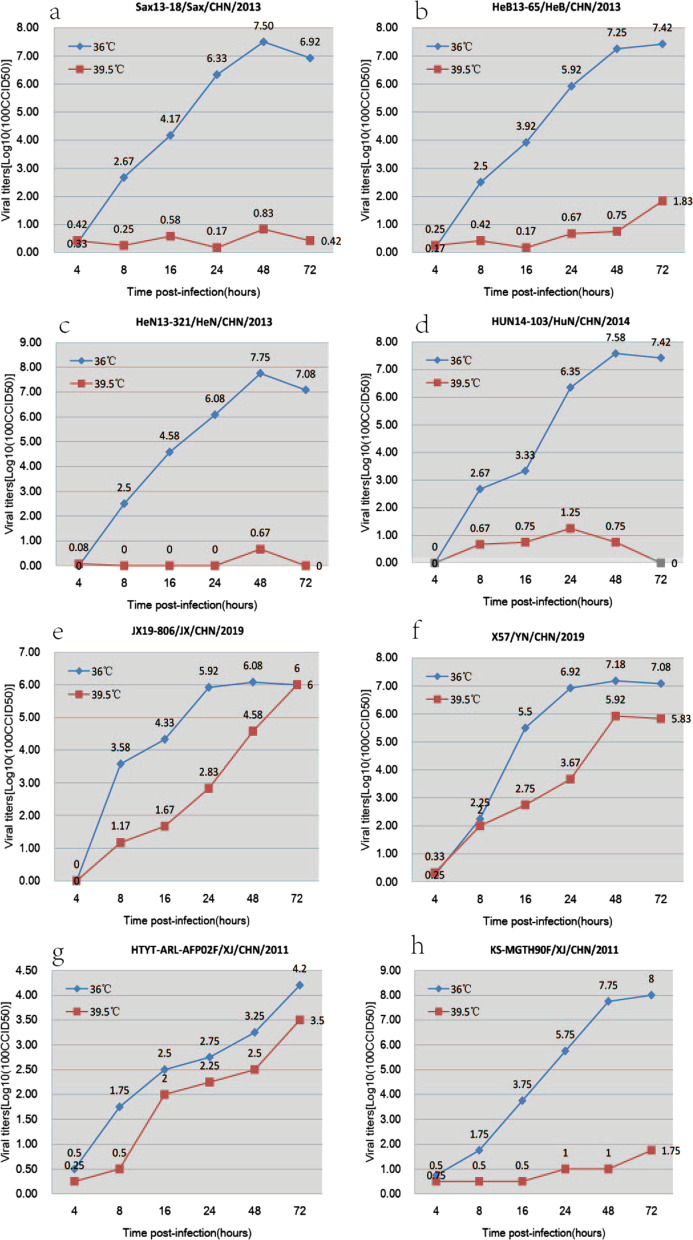
Temperature sensitivity test curves of the six representative Chinese E9 strains. Blue and orange lines represent the growth trends of the viruses on RD cells at 36 ℃ and 39.5 ℃, respectively. The Xinjiang EV-B85 strain (HTYT-ARL-AFP02F/XJ/CHN/2011, showing no temperature sensitivity) and the EV-B106 strain (KS-MGTH90F/XJ/CHN/2011, showing temperature sensitivity) were used as experimental controls

We selected six E9 representative stains from the fifteen E9 according to the Spatiotemporal separation distribution and genotypes to test the tolerance to the environment (Fig. 5). The results indicated that four strains (SaX-2013-18, HeB-2013-65, HeN-2013-321 and HuN-2014-103) were temperature sensitive and two others (JX19-806 and X57) were temperature resistant. The results showed that JX19-806 and X57 had higher tolerance to temperature compared to other strains.

The P1 region encodes the structural protein (vpl—vp4) of the virus and constitutes the capsid of the virus. In previous studies, capsid genes were shown to involve in determining the optimal growth temperature [30]. To investigate the possible mechanism underlying the difference in temperature sensitivity among these strains, we aligned nucleotide and amino acid substitutions in the P1 region. A total of 40 amino acid substitutions in the P1 region were summarized in Table 3. Comparison of amino acid substitutions showed that the temperature resistant strains have more amino acid substitutions than the temperature sensitive strains. Significantly, the amino acids at position 411, 865 and 867 in the temperature resistant strains had same mutations, whereas in the three temperature sensitive strains, the amino acid was same.

**Table 3:**
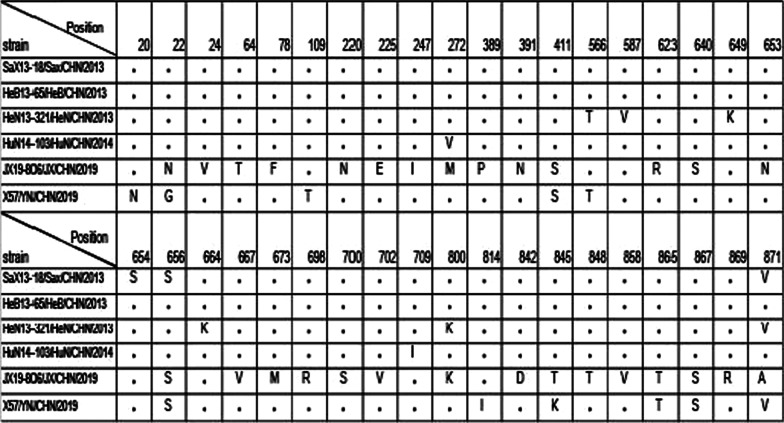
40 amino acid substitutions in the P1 region among the six E9 strains

The position of amino acid was calculated from the first amino acid of its P1 region after a multi-alignment by ClustalW

## Discussion

E9 virus has been circulating in worldwide for half a century, which endangers human health and increases the burden of disease. However, the molecular epidemiological study of E9 is very limited. We analyzed all available E9 sequence data in the word, including fifteen E9 genome-wide data obtained from the mainland of China in 2013–2019 in this study. Analysis of the origin and evolution of E9 indicated that the estimated mean evolutionary rate was 4.278 × 10^−3^ substitutions per site per year. In previous study, the mean evolutionary rate of EV71 was estimated to be 4.6 × 10^−3^ substitutions per site per year during its propagation process [31]. The mean evolutionary rate of E9 in worldwide was approximate to that of EV71, which hints the evolution of E9 is active. A burst of effective population size was observed in 1990s–2010s, then the effective population size is to a certain higher level than before and remained stable. Hence, we should be alert to the outbreak of E9 in the future.

D genotype of E9 is the dominant gene in Chinese mainland. The earliest strain of E9 D2 sub-genotype was isolated from Taiwan, China, we speculate that D2 sub-genotype of E9 circulating in mainland of Chinese was imported from Taiwan. Except for one strain isolated from Thailand, the remaining D3 sub-genotype of E9 was isolated from mainland of Chinese, we speculate that the Thailand strain of E9 was imported from mainland of Chinese. JX19-806 was the only C2 sub-genotype strain isolated from mainland of Chinese and its nucleotide sequence shows the highest similarities with the French strain isolated in 2012, which hints the E9 transmission along the border. As a lesson learned from these cases, in addition to the crucial route, domestic transmission, we should pay attention to the prevention of epidemics along the border.

Recombination is an important form of the evolution of enterovirus, and P2 and P3 were the main regions of recombination [32]. Analysis of the complete genome sequence of the fifteen E9 sequence data shows that E9 circulated in mainland of China had four recombination patterns. Recombination forms have no obvious time and regional characteristics. Recombination of E9 is characterized by universality and polymorphism.

It is very interesting that, among the six tested E9 strains, four (SaX-13-18, HeB-13-65, HeN-13-321 and HuN-14-103) were temperature sensitive and the other two (JX19-806 and X57) were temperature resistant. We aligned nucleotide and amino acid substitutions in the P1 region. According to the comparison of amino acid substitutions, we speculate that the amino acid substitutions at 411, 865 and 867 positions are related to changes in temperature-sensitivity. However, additional evidence is needed to support this hypothesis.

In conclusion, we analyzed the origin and evolution of E9 in worldwide. It is indicated that more attention should be paid for the prevention of epidemics along the border and the outbreak of E9 in the future. We reported the full-length genome sequences of fifteen E9 strains isolated from the mainland of China in 2013–2019. Sequence analysis suggested that these fifteen E9 strains have high genetic diversity compared with Barty strain, suggesting recombination within the non-structural protein-encoding region, and extensive genetic exchange with other EV-B serotypes, such as CVB5, E3, E11, EV-B106, and EV-B86. Some of the isolated E9 strains were temperature resistant. Hence, E9 has the potential to become a more common strain. This study provides valuable information regarding the molecular epidemiology of E9.

Although this study has included all E9 available sequences worldwide, this study is still lack of sequences isolated in certain regions and time periods. This may bias the bioinformatics analysis. With the coverage of the E9 sequence, the bioinformatics analysis will include sequences with a larger time and regional distribution span, and the results will be more accurate and representative.

## Conclusion

This study highlights a persistent transmission network of E9 in worldwide, provides valuable information regarding the molecular epidemiology of E9.

## Data Availability

Condensed anonymized data are available from the corresponding author on reasonable request.

## Abbreviations

E9: Echovirus 9
RT-PCR: Reverse transcription polymerase chain reaction
CI: Confidence interval
EV-B: *Enterovirus* B
AM: Aseptic meningitis
HFMD: Hand, foot, and mouth disease
AFP: Acute flaccid paralysis
NIVDC: National Institute for Viral Disease Control and Prevention
RD: Rhabdo-myosarcoma
Hep-2: Human larynx carcinoma
MCMC: Markov Chain Monte Carlo
GTR: General Time Reversible
G4: Gamma distributed rate with four rate catagories
ESS: Effective sample size
MCC: Maximum clade credibility
RGD: Argnine-glycine-aspartic acid
